# Glial connexins in glaucoma

**DOI:** 10.3389/fnins.2025.1560344

**Published:** 2025-04-09

**Authors:** Qiuyi Song, Xi Wu, Jiawei Yang, Siqi Li, Junguo Duan

**Affiliations:** ^1^Chengdu University of TCM, Chengdu, China; ^2^Eye College of Chengdu University of TCM, Chengdu, China; ^3^Key Laboratory of Sichuan Province Ophthalmopathy Prevention & Cure and Visual Function Protection with TCM Laboratory, Chengdu, China; ^4^Ineye Hospital of Chengdu University of TCM, Chengdu, China

**Keywords:** glial connexins, glaucoma, neuroinflammation, Cx43, gap junction

## Abstract

Glial cells play a crucial role in maintaining central nervous system (CNS) homeostasis and facilitating the repair of neural tissue following injury. The regulation of neuroglia may serve as a safe and effective strategy for modulating neuroinflammatory responses and restoring glial homeostasis and defense functions. Given that the glial network is composed of connexin (CX) proteins, its neuroprotective role is extensive. Therefore, connexins should be considered as functional “bridges” within this network. This review examines evidence for the active involvement of glial networks in neuroinflammation under both physiological and pathological conditions and summarizes the role of CXs in glaucoma. Finally, potential therapeutic strategies for glaucoma are explored.

## Introduction

Neuroglia, represented by astrocytes, oligodendrocytes, and microglia, are essential for maintaining the stability of the CNS (Tezel, 2022). They are crucial for preserving the structural integrity of the retina and serve as key regulators of retinal cell metabolism (Yoo et al., 2021). Pathological stimulation of the CNS, such as neuronal injury or various forms of damage, can activate glial cells. Reactive glial proliferation, observed in nearly all retinal diseases, supports retinal neuron survival but may also accelerate neuronal degeneration (Ou et al., 2022). These processes are associated with CXs on glial cells, which form highly interconnected networks through gap junctions (GJs) or hemichannels (HCs; Giaume et al., 2021). They maintain neuronal function under physiological conditions but accelerate disease progression under pathological conditions (Giaume et al., 2021; Huang et al., 2021). Substantial evidence has demonstrated the role of GJs and HCs in various CNS diseases, including but not limited to neurodegenerative disorders, with Cx43 in particular implicated in a wide range of neuropathologies (Charvériat et al., 2021). For instance, sites of enhanced Cx43 immunoreactivity have been identified in patients with Alzheimer's disease (AD) and in animal models of amyotrophic lateral sclerosis (ALS) and Parkinson's disease (PD; Provenzano et al., 2023; Maldonado et al., 2022; Seo et al., 2021). Inhibitors of glial cell CXs have been extensively studied over recent decades and have been found to differentially downregulate CXs expression in neurodegenerative diseases, chronic pain, epilepsy, and demyelinating disorders. These inhibitors regulate channel switching and suppress glial cell activation, thereby protecting neurons (Giaume et al., 2021; Charvériat et al., 2021).

In recent years, several studies have demonstrated that glial cell-mediated neuroinflammation influences the pathophysiology of glaucoma (Hu et al., 2021; Mélik Parsadaniantz et al., 2020). Glial cells are highly interconnected through CXs and function as an extensive cellular network (Boal et al., 2021). Therefore, this review explores the role of glial protein CXs in the neuroinflammatory response to glaucoma, extending beyond a focus on astrocytes as individual cellular units to provide insights for future research and therapeutic strategies.

## Glaucoma: a neurodegenerative disease

Glaucoma, a primary cause of irreversible blindness worldwide, is characterized by progressive optic nerve damage and visual field loss (Quigley, 1999). Approximately 80 million people are affected by the disease, and this figure is projected to surpass 100 million by 2040 (Tham et al., 2014; Bourne et al., 2013). Clinically, glaucoma is categorized into open-angle glaucoma (OAG) and closed-angle glaucoma (ACG), based on the configuration of the anterior chamber angle. Current clinical treatments primarily focus on reducing intraocular pressure (IOP); however, despite effective IOP control, ~15%−25% of patients continue to experience progressive optic nerve damage (Soto and Howell, 2014; Tezel, 2022).

Mechanical and vascular factors are believed to contribute to the etiology of glaucoma (McMonnies, 2018). The pathogenic mechanisms of glaucoma are complex and not yet fully understood. Recent studies suggest that glaucomatous optic neuropathy is a neurodegenerative disorder sharing common neuroinflammatory mechanisms with classical neurodegenerative diseases (Mélik Parsadaniantz et al., 2020). The CNS, which includes the brain, retina, and optic nerve, is an immune-privileged tissue (Lampron et al., 2013). Neurodegenerative diseases exhibit specific mechanisms that induce inflammatory responses, such as the production of inflammatory inducers, the activation of innate immune cells in the CNS as sensors of neurological damage, and the amplification of key regulated transcription factors, leading to inflammatory responses, neurotoxicity, and neuronal death (Teleanu et al., 2022; Voet et al., 2019). In contrast to classical inflammation, glial-driven neuroinflammation exists in an intermediate state known as para-inflammation (Medzhitov, 2008). While this state may initially represent a beneficial response, it can subsequently progress into a neurodegenerative process (Bariş and Tezel, 2019).

## Glial activation-mediated neuroinflammation

Astrocytes and microglia are resident immune cells that perform innate immune functions within the retina and optic nerve (Salkar et al., 2024). Astrocytes play a crucial role in maintaining tissue homeostasis within the retina and optic nerve. Under physiological conditions, astrocytes form networks through CXs to sustain the homeostatic environment necessary for neuronal function (Lopez Ortiz and Eyo, 2024). However, studies have indicated that excessive activation of astrocytes can contribute to retinal ganglion cell (RGC) damage (Boal et al., 2021). Reactive astrocytes mediate extracellular matrix remodeling and pro-fibrotic processes, ultimately accumulating within the optic nerve head, thereby increasing glaucoma-related biomechanical and vascular pressures and promoting RGC apoptosis (Burgoyne, 2011).

Microglia are tissue-resident immune cells which are crucial for initiating inflammatory responses. These cells continuously monitor and maintain homeostasis through clearance and phagocytosis, while also providing neurotrophic support (Borst et al., 2021). They can switch between M1 (pro-inflammatory) and M2 (anti-inflammatory) phenotypes in response to environmental stimuli (Orihuela et al., 2016). They exhibit sensor and effector functions, as well as phagocytic activity, under both physiological and pathological conditions (Borst et al., 2021). Like macrophages, hyperactivated microglia can promote neuroinflammation. This process may be associated with the secretion of pro-inflammatory cytokines, such as interleukin-6 (IL-6) and tumor necrosis factor-alpha (TNFα), as well as reactive oxygen species (Woodburn et al., 2021). The proliferation of reactive microglia has also been observed in experimental glaucoma models (Fernandez-Albarral et al., 2022).

Microglia in the glaucomatous retina often demonstrate a mixed activation phenotype. M2 microglia are activated only in the early stages of elevated IOP, whereas M1 microglia remain continuously activated throughout sustained elevated IOP. In experimental models of glaucoma, elevated IOP results in the differentiation of most microglial cells into an M1 phenotype, while only a few M2 microglial cells are identified within RGCs (Wei et al., 2019). In the chronic ocular hypertension retina, ATP-induced microglial activation stimulates the release of pro-inflammatory factors (Xu et al., 2022). Additionally, ATP transiently increases the mRNA levels of anti-inflammatory factors in microglia. However, the protein concentrations of these anti-inflammatory factors remain relatively low, resulting in negligible neuroprotective effects of activated microglia (Hu et al., 2021). These findings suggest that while activated microglia may provide some neuroprotection in the early stages of glaucoma, their activation predominantly contributes to RGC damage by exacerbating neuroinflammation.

## Glial connexins in neuroinflammation

Glial cells play a crucial role in maintaining CNS homeostasis and facilitating repair following injury. Their neuroprotective function is extensive due to the presence of glial GJs, which are composed of CXs (Caruso et al., 2023; Denaro et al., 2025). CXs constitute a multigene family, with 21 members identified in humans (Danish et al., 2021). The expression patterns of Cx proteins in various types of glial cells are summarized in Table 1.

**Table 1:** Expression of connexins in glial cells.

**Cell type**	**Primary connexins expressed**	**Other connexins expressed**	**References**
Astrocytes	Cx43, Cx30	Cx26 (in specific regions)	Altevogt and Paul, 2004; Boal et al., 2021
Oligodendrocytes	Cx29, Cx32, Cx45, Cx47	Panx1 (activated under stress or disease conditions)	Dermietzel et al., 1997
Microglia	Cx43 (low expression under basal conditions)	Cx36, Cx32, Cx45 (upregulated upon activation)	Eugenín et al., 2001; Garg et al., 2005
Müller cells	Cx43 (expression varies by animal model)	Cx30, Cx45, and Cx46 mRNA (detected in subsets)	Boal et al., 2021; Kerr et al., 2010; Söhl et al., 2000; Voigt et al., 2015

CXs exhibit unique physiological and pathological functions across different subtypes. Cx43, expressed in astrocytes, RGCs, and TM, regulates GJs, HCs, ATP release, and inflammatory responses, impacting neuroinflammation and ECM remodeling (Chen and Tang, 2022; Tellios et al., 2019; Sui et al., 2021). Cx30, in astrocytes and Müller cells, modulates synaptic plasticity, glutamate regulation, and neuroprotection (Giaume et al., 2021; Li et al., 2025). Cx32, in oligodendrocytes and Schwann cells, supports myelin maintenance and potassium buffering, with mutations causing X-linked Charcot-Marie-Tooth disease (Kleopa, 2011; Sargiannidou et al., 2009; Oguro et al., 2001). During the prenatal stage in mice, Cx26 has been reported to be expressed in neural progenitor cells, where it is primarily associated with neuronal migration and the development of neocortical circuits (Oguro et al., 2001). Cx36 is a neuron-specific Cx capable of forming functional channels. Cx45 has been observed to exhibit immunoreactivity in some MAP2 neurons and non-neuronal cells (Condorelli et al., 2003). Cx47, also in oligodendrocytes, facilitates oligodendrocyte-astrocyte communication, with dysfunction associated with leukodystrophies and multiple sclerosis (Nutma et al., 2020). These isoforms collectively underpin glial and neuronal function, with their dysregulation contributing to neurodegenerative and neuroinflammatory disorders.

**Figure 1:**
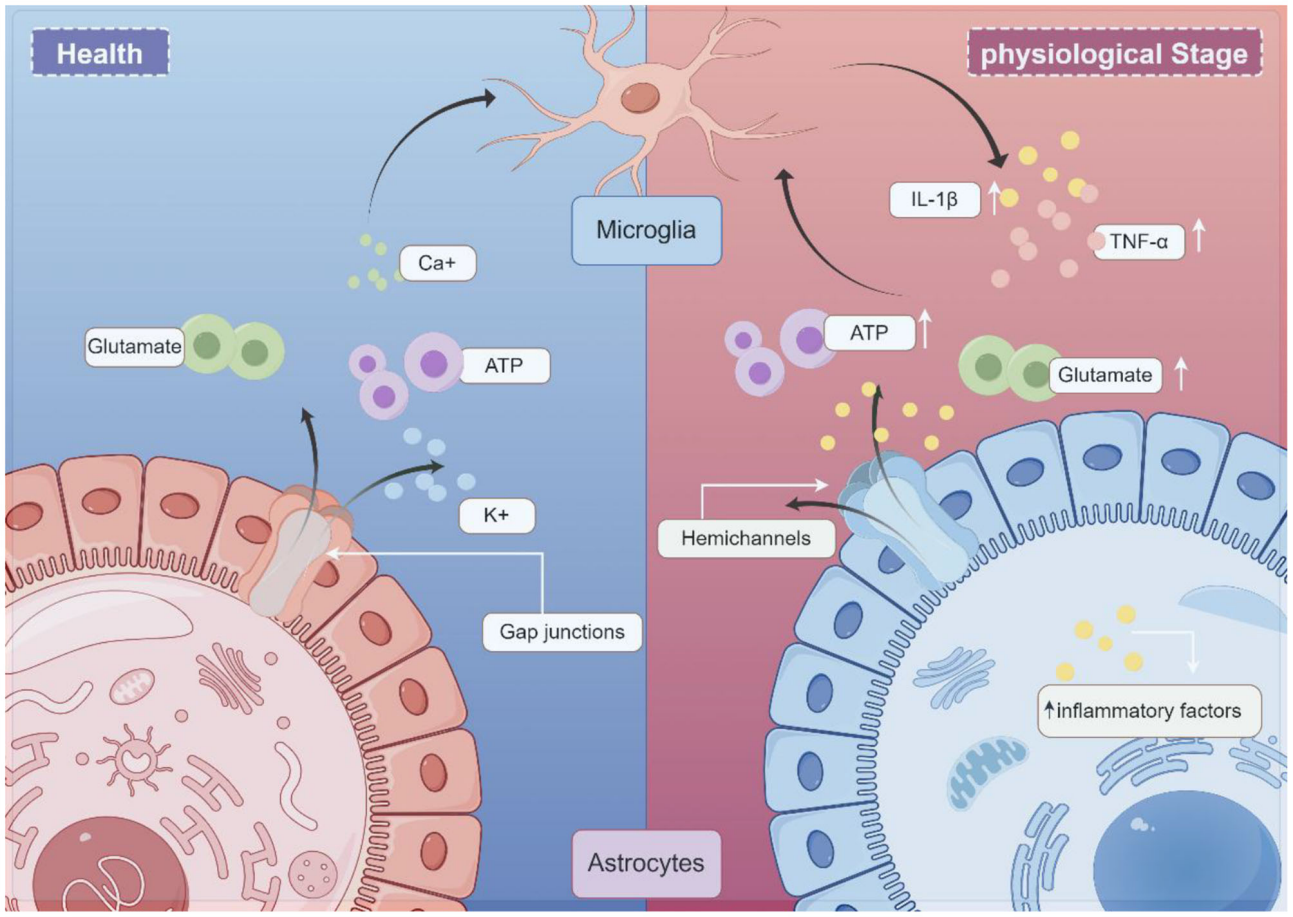
Schematic illustration of the working model glial CX in neurologic disease (draw by Figdraw). Under normal physiological conditions, gap junctions (GJs) allow the exchange of molecules between cells. These permeable molecules include ions (Ca2+, K+), nutrients (e.g., glucose), signaling molecules (e.g., ATP), and the neurotransmitter glutamate. Under pathological conditions, astrocytes and microglia become activated. Microglia secrete proinflammatory cytokines, such as IL-1β and TNF-α, and subsequently activate hemichannels (HCs). Excessive ATP and glutamate release by astrocytes, in turn, act on microglia, initiating an inflammatory cascade.

HCs are transmembrane channels consisting of six Cx subunits. Two hemichannels can pair to create a complete GJ, enabling direct communication between adjacent cells. However, when hemichannels remain isolated within the cell membrane, they are referred to as “hemichannels” or “connexons,” facilitating molecular exchange between the intracellular and extracellular environments. HCs primarily facilitate the transfer of ions and small molecules between these compartments (Bennett et al., 2003; Lei et al., 2023; Bennett et al., 2012). A single GJs is composed of two HCs or connexons, each formed by six CXs subunits. Moreover, glial cell communication occurs either directly through GJs or over short distances via autocrine/paracrine signaling through HCs. GJs permit the intercellular exchange of various molecules, including ions (Ca^2^^+^, K^+^), essential nutrients such as glucose, signaling molecules such as ATP, cAMP, and IP3, as well as the neurotransmitter glutamate (Anderson and Swanson, 2000; Harris, 2007; Vinken, 2015; Figure 1).

Astrocytes play a crucial role in the neuroinflammatory response (Huang et al., 2021). The glial network requires further investigation due to its significance in this process. In general, GJs are believed to exert various neuroprotective functions. From both metabolic and buffering perspectives, GJs contribute to neuroprotection by redistributing nutrients to damaged regions and regulating the extracellular environment. However, the neuroprotective effect of GJs appears to be condition-dependent. GJs have been observed to close under extreme conditions, such as high intracellular Ca^2^^+^ concentrations, to prevent the propagation of death signals through the astrocytic network (De Vuyst et al., 2009). This response may be associated with the duration and severity of inflammatory damage. HCs play a pivotal role in inflammatory conditions, exhibiting contrasting pathophysiological roles compared to GJs. Often regarded as “pathological channels,” HCs exhibit pro-degenerative properties in pathological conditions. During neuroinflammation, activated microglia and astrocytes lead to the secretion of IL-1β and TNF-α pro-inflammatory cytokines by microglia, subsequently opening HCs. This exacerbates neuro-excitotoxicity through excessive ATP and glutamate release, perpetuating a cycle of chronic inflammation (Denaro et al., 2025). These findings suggest that Cx proteins play a pivotal role in neuroinflammation.

In the EAE (experimental autoimmune encephalomyelitis) animal model of multiple sclerosis, conditional knockout of Cx43 resulted in a reduction in the expression of pro-inflammatory cytokines IL-6, interferon-γ, and IL-10, leading to attenuated inflammatory responses and decreased demyelination. These findings suggest that knockout of Cx43 promotes astrocyte polarization toward an anti-inflammatory phenotype and ameliorates EAE pathogenesis by lowering pro-inflammatory cytokine and chemokine concentrations in the cerebrospinal fluid (Une et al., 2021). Additionally, studies on Cx30 knockout (KO) EAE mice revealed extensive microglial activation during the chronic phase of EAE. Furthermore, Cx30 deficiency facilitated astrocyte activation and promoted the differentiation of neuroprotective microglia, thereby mitigating chronic neuroinflammation in EAE (Fang et al., 2018).

Research on the role of microglia in neuroinflammation is also increasingly abundant. Studies have shown that the conditional knockout of Cx43 in EAE promotes the expression of M2. During the pre-immunization phase, Cx43 icKO spinal microglia demonstrate increased expression of anti-inflammatory genes while suppressing pro-inflammatory gene expression, thereby establishing an anti-inflammatory state prior to immunization. This, along with their synergistic interaction with astrocytes, contributes to the attenuation of EAE progression (Wang et al., 2024). Notably, in mouse models of glaucoma, the inhibition of GJs or ablation of Cx36 has been demonstrated to prevent dendritic remodeling and RGC death (Kumar et al., 2023). It is hypothesized that this phenomenon may be associated with the phenotypic switching of glial cells, which warrants further investigation. Collectively, these findings suggest that Cx proteins play a pivotal role in neuroinflammation.

In the CNS, glial cells, particularly astrocytes, predominantly express Cx43 (Giaume et al., 2021). Growing evidence supports the involvement of astrocyte Cx43 HCs in mediating neuroinflammatory processes (Retamal et al., 2007). Under inflammatory conditions, the activation of inflammasome pathways may coincide with increased Cx43 expression, thereby sustaining abnormal Cx43-mediated ATP release. As pro-inflammatory mediators, HCs facilitate the transfer of toxic metabolites during neuroinflammatory responses. Notably, Cx43 HCs significantly contribute to the amplification and persistence of inflammation by mediating the ATP autocrine feedback loop within the inflammatory cycle (Chen and Tang, 2022).

Collectively, these studies indicate that Cx43 HCs play a crucial role in neuroinflammatory processes. Therefore, strategies targeting glial Cx43 HC activity represent a promising therapeutic approach for neuroinflammatory diseases requiring novel treatment options, as suggested by findings on Cx43 deficiency and pharmacological blockade in neuroinflammation (Yin et al., 2018; Wang et al., 2021, 2020).

## Connexin43 channel modulation

As a predominant connexin following glial activation, Cx43 is regulated by a variety of signaling pathways and, in turn, modulates downstream signals essential for neuronal activity, contributing to various central nervous system diseases, including glaucoma. Current gap junction modulators include global non-specific blockers, connexin isoform-specific antisense oligonucleotides, and connexin mimetic peptides (Becker et al., 2016).

Global non-specific blockers indiscriminately affect both GJs and HCs. Commonly used compounds include glycopyrrolate derivatives (Davidson et al., 1986; Takeuchi et al., 2011), as well as anesthetics (Mantz et al., 1993) and anti-inflammatory agents such as enflurane, isoflurane, flufenamic acid, niflumic acid, and meclofenamic acid (Harks et al., 2001; Srinivas and Spray, 2003). Antisense oligonucleotides enter the cell and bind to messenger RNA (mRNA), thereby inhibiting protein synthesis. Their advantages include high specificity and the ability to enable rapid restoration of function. Additionally, their long half-life allows for sustained delivery. Notably, experimental evidence suggests that antisense oligonucleotide-mediated attenuation of TGFβ signaling can restore neural function in adult non-human primates (Peters et al., 2021). Connexin mimetic peptides are short peptide sequences designed to mimic specific intra- and extracellular regions of connexin proteins. Gap26, Gap27, and Gap19 have been widely utilized to inhibit gap junctions primarily composed of Cx43 (Lucero et al., 2020; Li et al., 2015; Walrave et al., 2016). Gap26 attenuates LPS-induced apoptosis by inhibiting the PI3K/AKT pathway (Liu et al., 2021). The application of Peptide 5 effectively reduced Cx43 levels and the number of glial fibrillary acidic protein (GFAP)-positive astrocytes while mitigating neuronal loss in a concentration- and time-dependent manner. Interestingly, low concentrations of peptide 5 prevented HC opening but preserved GJ communication, whereas higher concentrations prevented HC opening but led to the uncoupling of existing GJs (O'Carroll et al., 2008). These findings suggest that connexin mimetic peptides can selectively inhibit HCs while maintaining GJ functionality in a dose- and time-dependent manner.

Therefore, given the critical role of gap junction channels (GJCs) and HCs in glial activity, CXs represent a promising therapeutic target for glaucoma treatment. CX43-mediated glial networks have been proposed as a potential therapeutic approach for glaucoma.

## Connexin43 in neuroinflammation of glaucoma

**Figure 2:**
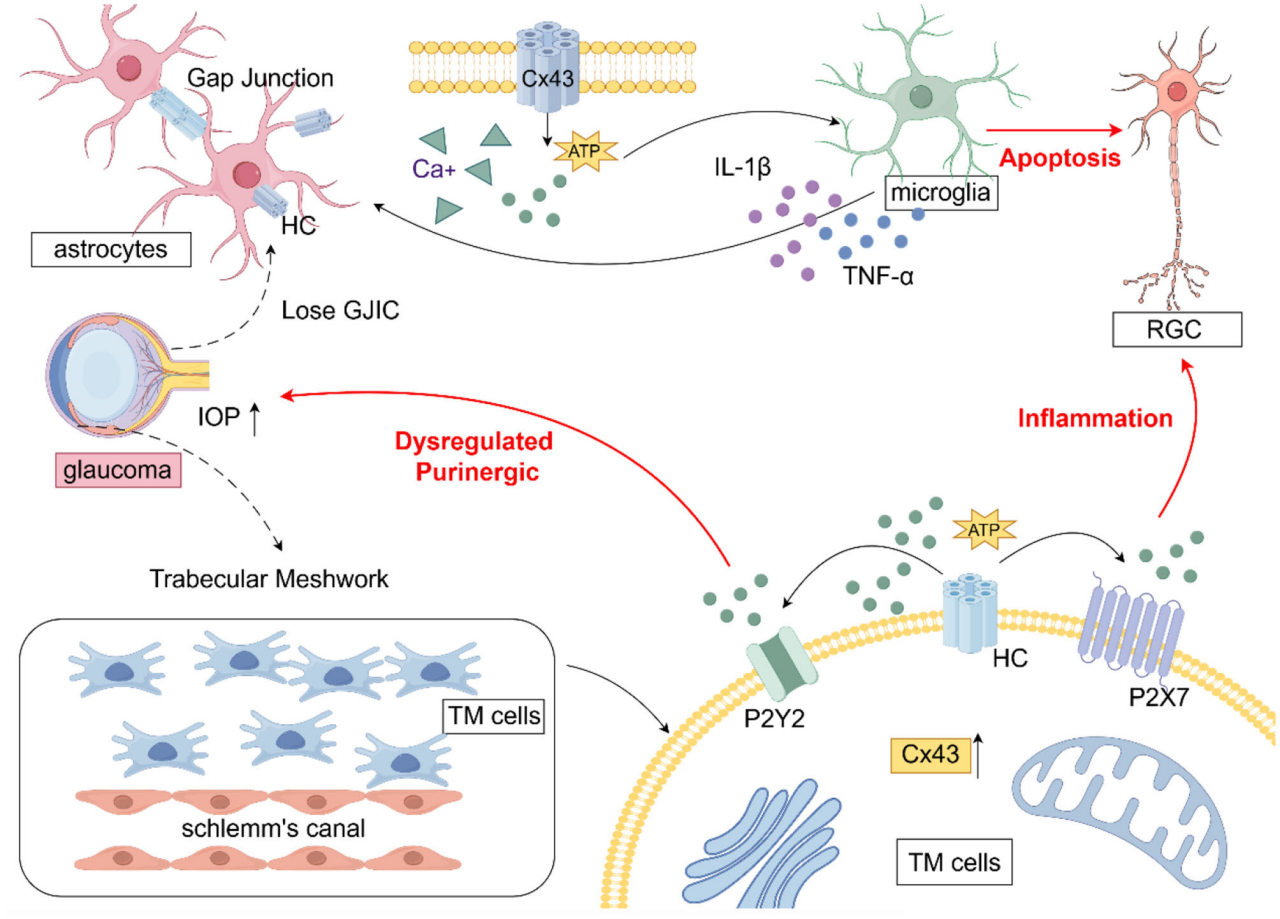
The role of CX43 in glaucoma (draw by Figdraw). In glaucoma, astrocyte activation leads to the upregulation of Cx43, which disrupts gap junction intercellular communication (GJIC) and facilitates hemichannel (HC) opening, resulting in excessive ATP release. This ATP release promotes the transition of microglia to a pro-inflammatory phenotype, triggering the secretion of cytokines that further exacerbate astrocyte activation, ultimately resulting in retinal ganglion cell (RGC) apoptosis. Within trabecular meshwork (TM) cells, the upregulation of Cx43 and the subsequent ATP release disrupt purinergic signaling homeostasis through P2Y2 receptors, contributing to increased IOP.

Studies have shown that connexins, particularly CX43, are closely associated with glaucoma. CX43 is widely expressed in the trabecular meshwork (TM) and is predominantly found in astrocytes, Müller cells, and microglial cells, with the highest prevalence in astrocytes (Boal et al., 2021; Tellios et al., 2019). Figure 2 illustrates the role of CX43 in glaucoma. Studies have shown that upregulation of CX43 serves as an initial trigger for the inflammatory cascade leading to RGC death following ischemic retinal injury. However, blocking CX43 HCs can preserve the integrity of the blood-retinal barrier, thereby reducing glial-related secondary tissue damage and exerting significant neuroprotective effects (Toychiev et al., 2021). A well-established causal link exists between vascular dysfunction and glial inflammation. The involvement of Cx43 HCs in RGC death in glaucoma cannot be ruled out. Strong evidence suggests that glaucoma is associated with changes in astrocytic Cx43 expression in both the retina and optic nerve.

Cx has been implicated in glaucoma progression through its modulation of neuroinflammation and alterations in gap junction intercellular communication (GJIC). Astrocytes, the predominant glial cells in the optic nerve head, form functional syncytia via Cx43-mediated gap junctions, facilitating communication and maintaining ionic and metabolic homeostasis in RGC axons. Elevated IOP in glaucoma has been associated with the loss of GJIC in astrocytes, disruption of RGC axonal homeostasis, acquisition of a reactive phenotype, and promotion of glaucomatous neurodegeneration (Malone et al., 2007). Upregulation of astrocytic Cx43 has been shown to enhance the release of pro-inflammatory factors, exacerbating neural damage. Notably, blocking GJs or ablating Cx36 has been demonstrated to prevent dendritic remodeling and RGC death in murine glaucoma models (Kumar et al., 2023). In glaucoma, increased CX43 immunoreactivity has been observed in the lamina cribrosa, peripapillary, and midperipheral retina, correlating with glial activation (Kerr et al., 2011). In human glaucoma optic nerve head astrocytes, Connexin43 gene expression was found to be elevated compared to controls. CX43 has been reported to mitigate neuroinflammation and alleviate neurodegenerative stress through metabolic resource redistribution in a mouse model of elevated IOP (Cooper et al., 2020). In an experimental glaucoma model with CX43 genetic ablation, astrocytic Cx43 deletion was found to be neuroprotective, enhancing RGC survival (Batsuuri et al., 2021). Astrocytic regulation of glaucomatous RGC survival has been linked to Cx43-mediated ATP release, which may involve modulation of the Rac1/PAK1/CX43 pathway and could represent a potential neuroprotective strategy in glaucoma (Zhao et al., 2021). Additionally, in animal models of unilateral optic nerve injury, retinal Cx43 protein levels were upregulated in both eyes, including the uninjured eye, in parallel with astrocyte activation. These findings suggest that the CX43 network may contribute to the propagation of glaucoma pathology (Chew et al., 2011).

Cx43 is widely expressed in GJs of the TM, where it regulates intercellular communication and extracellular matrix remodeling through both GJs and HCs, thereby influencing aqueous humor outflow and IOP regulation. The upregulation of Cx43 in damaged TM has been identified as a novel phenomenon, suggesting its involvement in the pathogenesis of glaucoma and its significant functional role in this process (Tellios et al., 2019). In a study utilizing a glaucoma mouse model to investigate the role of Cx43 in TM functional regeneration, knockdown of Cx43 expression was shown to attenuate the transplantation of induced pluripotent stem cell (iPSC)-derived TM cells, hinder TM function recovery, suppress endogenous TM cell division, and prevent the restoration of disrupted IOP homeostasis in glaucoma (Sui et al., 2021). *In vitro* studies have shown that mechanical stretch, simulating elevated IOP, induces upregulation of astrocytic Cx43 expression and promotes apoptosis (Tellios et al., 2019). This effect may be attributed to stress-induced Cx43 upregulation, which enhances intercellular communication and ATP efflux through these channels.

CXs have traditionally been recognized as transmembrane proteins that form gap junctions to facilitate intercellular communication. Recent studies have demonstrated that CXs influence tissue homeostasis through channel-independent mechanisms (Ha, 2009). The non-channel-dependent functions of CXs are integral to transcription, metabolism, cell/cell and cell/extracellular matrix (ECM) adhesion, and cell signaling (Iacobas et al., 2004; Li et al., 2023).

Cx43 has been localized to mitochondria in various cell types, including astrocytes (Kozoriz et al., 2010). Studies indicate that mitochondrial Cx43 (mtCx43) regulates ATP and ROS production, thereby modulating tissue injury, inflammation, and apoptosis (Li et al., 2023). Mitochondrial dysfunction and oxidative damage in RGCs are central mechanisms in glaucomatous neurodegeneration. Thus, mtCx43 dysfunction may exacerbate apoptosis in this condition. Another study demonstrated that Annular Cx43 HC-containing vesicles can form Cx43 GJs with recipient cells, serving as a mechanism for facilitating internalization that modulates inflammatory responses and degenerative processes (Varela-Eirín et al., 2022), potentially contributing to glaucoma progression.

In the TM, Cx43 interacts with the scaffolding protein ZO-1 and associates with integrins, forming multi-protein complexes that regulate ECM remodeling in response to mechanical stress (Li et al., 2020a). Another study identified Cx43 as a key regulator of phagocytic activity in human TM cells, thereby contributing to glaucoma pathogenesis (Li et al., 2020b). Moreover, Cx43 has been implicated in the regulation of epithelial-mesenchymal transition (EMT) and fibrotic processes. Although the specific mechanisms require further investigation, these findings highlight CXs as promising therapeutic targets for these disorders (Li et al., 2023).

These non-channel functions of CXs offer novel insights into glaucoma pathophysiology. Future research should utilize glaucoma models to elucidate regulatory mechanisms and explore therapeutic potential in neuroprotection and intraocular pressure regulation.

Therefore, targeting Cx43 may simultaneously improve aqueous humor dynamics and provide neuroprotection, potentially representing a novel approach for the comprehensive management of glaucoma. It is evident that the pathological mechanisms of HCs and GJs play a crucial role in disease progression. The gap junction network may exert neuroprotective effects during the early stages of the disease. Given that glaucoma is a chronic progressive condition, the roles of HCs and GJs in its pathogenesis warrant further investigation.

## Summary and outlook

CX43 in glial cells ensures proper glial function and neuronal activity, which, in turn, regulates their survival and activity. This coordination is essential for maintaining functional integration throughout the CNS. GJs and HCs, formed by CXs, are both functionally synergistic and antagonistic. The role of HCs as pathological channels in glial activation is now well-established, as HCs become activated in pathological conditions, regulating various downstream signaling factors that accelerate neuroinflammation and promote disease progression. However, the role of GJs remains uncertain, as they may exert both beneficial and deleterious effects. Further clarification is required regarding the regulatory nodes that mediate these opposing effects. Additionally, the signaling pathways of connexins in neuroprotection warrant further investigation.

A better understanding of the complex role of astrocyte CX43 in glaucoma may facilitate significant therapeutic advancements. However, CX43 is widely expressed in multiple tissues throughout the body, posing a considerable challenge for the specific targeting of ocular CX43. Localized drug delivery and gene therapy techniques may enhance treatment specificity and safety. Studies have demonstrated that excessive opening of CX43 hemichannels is associated with ganglion cell damage in glaucoma. The use of specific inhibitors (such as Gap19 and TAT-Gap19) can mitigate neuroinflammation and apoptosis, highlighting their potential for clinical application. Regulating CX43 expression through gene-editing technologies (e.g., CRISPR/Cas9) or RNA interference (siRNA) may also provide innovative therapeutic avenues for glaucoma. Additionally, developing peptides that mimic CX43 function to modulate intercellular communication and protect retinal ganglion cells represents another promising strategy. Furthermore, utilizing carriers such as nanoparticles or liposomes to precisely deliver CX43-related drugs to ocular targets may serve as a novel therapeutic approach for glaucoma management. More refined clinical evaluation methods are required to accurately assess the efficacy of CX43-targeted therapies in glaucoma patients. Currently, most CX43-related therapeutic strategies are still in the preclinical phase, and future large-scale clinical trials are required to validate their safety and effectiveness. In summary, CX43 represents a promising therapeutic target for glaucoma, with multiple strategies under development. As research advances and technology progresses, CX43-based therapies are anticipated to emerge as a crucial component of future glaucoma treatment strategies.
